# Perspective: Moving Toward Desirable Linoleic Acid Content in Infant Formula

**DOI:** 10.1093/advances/nmab076

**Published:** 2021-07-15

**Authors:** Susan E Carlson, Lidewij Schipper, J Thomas Brenna, Carlo Agostoni, Philip C Calder, Stewart Forsyth, Philippe Legrand, Marieke Abrahamse-Berkeveld, Bert J M van de Heijning, Eline M van der Beek, Berthold V Koletzko, Beverly Muhlhausler

**Affiliations:** Department of Dietetics and Nutrition, Kansas University Medical Center, Kansas City, KS, USA; Danone Nutricia Research, Utrecht, The Netherlands; Department of Pediatrics, University of Texas at Austin, Austin, TX, USA; Division of Nutritional Sciences, Cornell University, Ithaca, NY, USA; Pediatric Area, Fondazione IRCCS Ca’Granda- Ospedale Maggiore Policlinico, Ca' Granda, Ospedale Maggiore Policlinico, Milan, Italy; Department of Clinical Sciences and Community Health, University of Milan, Milan, Italy; Faculty of Medicine, University of Southampton, Southampton, United Kingdom; University of Dundee, West Ferry, Dundee, United Kingdom; Laboratoire de Biochimie-Nutrition Humaine, Agrocampus–French National Institute of Health and Medical Research, Rennes, France; Danone Nutricia Research, Utrecht, The Netherlands; Danone Nutricia Research, Utrecht, The Netherlands; Danone Nutricia Research, Utrecht, The Netherlands; Department of Pediatrics, University Medical Center, Groningen, The Netherlands; Ludwig-Maximilians-Universität Munich, Department of Paediatrics, Dr von Hauner Children's Hospital, University of Munich Medical Center, Munich, Germany; Nutrition and Health Program, Health and Biosecurity, CSIRO, Adelaide, Australia; School of Agriculture, Food and Wine, The University of Adelaide, Adelaide, Australia

**Keywords:** linoleic acid, LCPUFAs, human milk composition, infant development, infant formula, nutritional programming

## Abstract

Infant formula should provide the appropriate nutrients and adequate energy to facilitate healthy infant growth and development. If conclusive data on quantitative nutrient requirements are not available, the composition of human milk (HM) can provide some initial guidance on the infant formula composition. This paper provides a narrative review of the current knowledge, unresolved questions, and future research needs in the area of HM fatty acid (FA) composition, with a particular focus on exploring appropriate intake levels of the essential FA linoleic acid (LA) in infant formula. The paper highlights a clear gap in clinical evidence as to the impact of LA levels in HM or formula on infant outcomes, such as growth, development, and long-term health. The available preclinical information suggests potential disadvantages of high LA intake in the early postnatal period. We recommend performing well-designed clinical intervention trials to create clarity on optimal levels of LA to achieve positive impacts on both short-term growth and development and long-term functional health outcomes.

## Teaser

**Statement of Significance**: The present work summarizes and discusses for the first time the (gaps in) available evidence on the effects of linoleic acid in an infant diet on infant health outcomes in relation to the recently adapted changes in regulations for the addition of linoleic acid to infant formula.

## Introduction

Exclusive breastfeeding is recommended for infants up to 6 months of age, with continued breastfeeding thereafter in conjunction with appropriate complementary feeding until 2 years of age or beyond (1). Lipids make up a substantial proportion of human milk (HM) macronutrients and provide around 50% of the total energy needs for the infant. The HM fatty acid (FA) profile is diverse, with over 200 FA species in varying isoforms and concentrations (2). The most abundant FAs in HM (>50% of the FAs) are saturated fatty acids, followed by MUFAs and PUFAs. PUFAs of various carbon chain lengths can be further classified as n–3 or n–6, depending on the location of the last double bond from the carboxyl end of the FA chain. HM n–3 and n–6 PUFAs include the essential fatty acids (EFAs) linoleic acid (LA; 18:2 n–6) and α-linolenic acid (ALA; 18:3 n–3), ranging between 5%–30% and 0.3%–2% of total FA, respectively, as reviewed previously (2, 3). However, these levels may vary depending on various maternal nutritional, lifestyle, and genetic factors.

LA and ALA are the predominant dietary PUFAs and the main substrates to produce the long-chain PUFAs (LCPUFAs), particularly arachidonic acid (ARA; 20:4 n–6) and DHA (22:6 n–3). Preclinical evidence suggests that preformed ARA and DHA may meet needs for EFAs, indicating that LA and ALA are not essential if sufficient ARA and DHA are provided (4). ARA and DHA are also present in HM, although at much lower levels than their precursors, with reported ranges between 0.1%–1.1% and up to 1.4% of the total FAs for ARA and DHA, respectively (2, 5), with the levels of DHA being largely dependent on the maternal diet (6–8). Though the relationship between the maternal dietary intake and HM concentration has not been determined for all PUFAs, the available evidence suggests that their wide range in HM reflects variations in maternal dietary intakes and maternal genotypes (9).

HM substitutes (i.e., infant formula) should provide a safe and nutritionally adequate alternative if full breastfeeding is not possible. Absent conclusive evidence on infant nutrient needs, knowledge of the physiology and composition of HM may provide some first guidance on designing the composition of infant formulas, along with scientific data from experimental and human studies on nutritional needs, safety, and biological effects.

While our knowledge of HM FA compositions and infant FA metabolisms has increased over the last several decades, new scientific insights and improved analytical techniques have highlighted the substantial variability in the concentrations of FAs in HM and the complex relationships among them. This suggests that requirements may vary among individual infants and complicates the formulation of infant nutritional guidelines. The variable fat content and FA compositions of weaning foods further affect overall FA intakes and our ability to assess the kinds and amounts of FAs infants need (10–12). A challenge for the formulation of guidelines on FA compositions in infant nutrition is the absence of reliable data on precise nutritional requirements for healthy infant growth and development and later-life health outcomes. The current narrative review paper summarizes an expert discussion on the topic initiated and organized by Danone Nutricia Research. It aims at providing an overview on the available evidence that may guide the definition of appropriate infant formula FA compositions, with an emphasis on LA.

## Infant FA Requirements

Nutrient levels in formula should ideally be adequate for all infants, while recognizing that optimal levels may not be the same for all, depending on genetic, other biological, and environmental factors. However, a challenge for the formulation of guidelines on PUFA intakes for infants is the limited existing data on the precise nutritional requirements for healthy growth and development and support of optimal long-term health outcomes. There is evidence that specific subgroups of infants have different FA requirements to the general infant population. For example, infants born preterm miss out on part of the in utero accumulation of DHA and ARA in their brain and other tissues that would normally accelerate during the third trimester, and have lower brain ARA and DHA at birth than infants born at term (13). Half of the brain DHA accretion at term occurs in the last 5 weeks of gestation (13–16). Preterm infants are at risk for neurodevelopmental problems and may benefit from ARA and DHA supplementation in the postnatal period (17–20), although there is limited evidence of beneficial effects on neurodevelopmental outcomes in the longer term (21). Compared to preterm and (very) low birth-weight infants, our understanding of the FA needs of infants born with a high birth weight or to women with obesity or diabetes is even more limited. Some evidence suggests that maternal and cord blood DHA concentrations are reduced in pregnancies complicated by maternal gestational diabetes (22), raising the possibility of an increased n–3 LCPUFA requirement postnatally.

It is likely that even among healthy, term infants there is a considerable variation in PUFA requirements based on phenotype, genotype, and (maternal) environmental factors. For instance, the FA metabolism is likely to be sexually dimorphic, given that sex differences in response to PUFA interventions have been reported in numerous infant studies (23). In addition, genetic variation in the FA desaturase (*FADS*) gene cluster determines the infant's capacity for endogenous synthesis of ARA and DHA from their precursors, and so may result in different nutritional needs and related disease risks (24). In support of this, children with the poor conversion phenotype (haplotype A compared with D) have a 2-fold higher risk of developing asthma if their DHA supply after birth is low (24, 25). Likewise, maternal diet, health, and lifestyle, as well as other environmental factors such as smoking, modulate the fetal FA supply and PUFA status at birth (26, 27), which suggests specific nutritional needs may arise for n–6 and n–3 LCPUFAs in the postnatal period.

Acknowledging individual variability in the FA status at birth, the requirements for specific PUFAs in formula may best be described as a range rather than a single value. Generally, it is assumed that the estimated average requirement plus/minus 2 SDs of the variation in infant requirements will cover the needs of almost all healthy individuals in a population. Crossing either side of the lower or upper margin could increase the risk of negative consequences for the infant's metabolism, physiological functions, and short- and long-term health outcomes. Although in theory this seems plausible, the current evidence is too limited to provide a strong rationale for clear cutoff values for specific FAs. As an example, adverse health effects were observed in clinical studies conducted in the 1940s and 1950s using experimental infant feeding concepts with very low LA levels (see also below). Although they provided valuable information on minimal requirements of LA (28), these studies were conducted using formulas with very low fat contents and devoid of preformed LCPUFAs, and are therefore not comparable to the infant formulas currently on the market.

In contrast, infant formula marketed in the Netherlands in the early 1970s provided as much as 58% of FAs as LA, resulting in very high LA contents in infant body fat (29), and an early study in the United States of infants fed formula with 45% of FAs as LA found 2- to 3-fold higher LA contents of red blood cell phospholipid classes compared to breastfed infants (30). No obvious adverse health effects were reported in these studies (30, 31). Clinical studies to investigate dietary levels of all FAs are impractical, if not impossible, and thus guidance is needed from careful consideration of scientific evidence, including an in-depth understanding of HM composition and preclinical studies.

## HM Composition May Provide Guidance on Adequate Infant FA Intakes

The nutritional and functional properties of infant formula should ensure healthy growth and development, serving a similar function and providing similar benefits as HM as much as possible. HM composition is believed to be selected by evolution to provide the nutrients and energy to support adequate growth and development of human infants (32), and thus provides some guidance as to infant FA intakes that are likely adequate, even though the HM lipid composition varies markedly. It is also important to consider that an identical intake level of a substrate in infant formula as observed in HM does not by itself ensure safety and suitability. For instance, the bioavailability of a given substance may differ between the formula and HM matrix and/or there may be subtle differences in the conformation of a substrate added to infant formula in comparison to the naturally occurring substance in HM (33). To date, recommendations for the lipid composition of infant formula have been guided partly by data on the lipid composition of HM (34), while acknowledging that exact mimicking of the HM composition may be neither possible nor favorable per se. For example, some components of HM might be present as a consequence of the mechanisms of HM synthesis, maternal diet, or other environmental factors, such as exposure to lipid-soluble pollutants without any nutritional or other benefit for the infant (35). Other components, such as hormones and other biologically active components present in HM, cannot be added to infant formula at this time because of sourcing, technological, regulatory, or food safety reasons.

More than 200 different FA species have been described in HM. For most, their relevance to the health and development of the infant is unknown. For some FAs, it is uncertain whether they are present in HM in all women. The variation in FA profiles in milk of healthy, omnivorous women could be used as a base to define appropriate ranges; however, several aspects need to be considered.

First, HM fat content and FA composition change considerably over the course of lactation (36, 37). It has been postulated that some of these changes may match changing nutritional requirements of the growing/developing infant. For example, the observed increase in fat content with a concomitant decrease of protein-to-fat and protein-to-energy ratios during the transition from colostrum to mature milk is likely to reflect the decreasing weight gain velocity with an increasing postnatal age (32). The determinants and biological relevance of changes in the FA composition of HM during the transition to mature milk, in particular the decline in HM ARA and DHA with an increasing duration of lactation, is less clear (2), and is considered to likely reflect the depletion of maternal body stores of LCPUFA rather than declining needs of the infant. With current concepts, the total fat content and FA composition of formula intended to be used by infants from birth up to 6 months of age is fixed according to 1 specific recipe, and even follow-on formulas for older infants often tend to have the same lipid composition, even though arguments for more differentiated staging of formula compositions have been brought forward (38). Hence, formula-fed infants currently lack the exposure to potentially functionally relevant changes in lipid composition that breastfed infants experience.

Second, the HM lipid composition is associated with various maternal factors, including lifestyle factors such as smoking, body composition, dietary patterns, *FADS* genotype, parity, and potentially child sex (9, 39–43). The extent to which these maternal factors impact the HM lipid composition varies among different FAs, and may also relate to the observed geographical variation in the HM lipid composition (5, 44). Previous investigations have provided strong evidence that the maternal dietary intake of DHA strongly affects the amount of this FA in HM (5–8, 45). Likewise, the maternal LA intake is related to the LA content of HM, although the predominant sources of LA in HM postpartum are maternal body fat stores (∼70%) rather than recent LA dietary intake (44, 46). This points to the relevance of the maternal diet prior to and during pregnancy, which modulates the LA content of maternal fat stores for the LA content in HM. In contrast to LA, the level of ARA in HM shows remarkably little variation in relation to differences in the maternal ARA intake (5).

Third, in addition to variations in the HM lipid composition as a result of differences in individual dietary intakes and other biological and lifestyle factors, marked temporal changes in HM FA composition have been documented at a population level, due to changes in habitual dietary intakes and food composition. During the 20th century, dietary intake of n–3 LCPUFAs declined, whereas the mean intake of LA markedly increased in Western/industrialized societies. This has been driven by increased use of vegetable oils that are rich in LA, such as soybean and sunflower oils, and the increased consumption of processed and baked food items that are rich in these oils, such as spreads, ready-to-use meals, and pre-prepared sauces (47–49). These dietary changes were reflected in markedly increased LA levels in the HM of Western women between 1944 and 2000 (3, 50). Moreover, considering more recent trends in the industrialized food supply, such as the replacement of high–LA vegetable oils by oils high in palmitic or oleic acids, such as palm oil, high-oleic sunflower oil, rapeseed (classic canola) oil, and olive oil, it may be postulated that the evolution of the HM FA composition will continue, i.e., become higher in oleic acid and lower in LA. A recent German study comparing HM FA compositions in samples collected from 2 cohorts a decade apart (2000 compared with 2012), however, suggests that the increase in HM LA levels continues and that the increased use of alternative oils is not (yet) visible at the HM level (51).

With regards to the selection of reference milk samples to be used as a guide to infant formula FA compositions, it is important to point out that *no* 2 HM samples are the same; there are considerable differences between, as well as within individuals. While pooling HM samples and data, thereby taking into account the aforementioned factors, can address this to some extent, it is impossible to account for it completely, especially given that HM compositions in part reflect patterns of edible fat consumption and the populations studied across the globe. Moreover, it should be considered that some of the variation in the FA content and composition of HM may not necessarily translate into favorable effects for the developing infant. Furthermore, in addition to the FA content, differences in fat digestion and digestibility between infants, including the lipase activity and triglyceride structure affecting FA absorption, have to be considered, as well as other effects related to differences in the mode of feeding (formula feeding compared with breastfeeding), which also have the potential to influence the FA uptake and HM composition (52, 53). Hence, taking the large inter- and intra-individual variations in HM FA compositions into account, it is clear a single, ideal HM composition does not exist as a guidance/reference to define appropriate levels of FAs for a population-based adequate intake.

## Current Recommendations for Infant Formula Lipid Composition

Recommendations set by the Codex Alimentarius (“Codex”) of the FAO and WHO on formula composition state that the fat content in infant formulas should be between 4.4 and 6.0 g/100 kcal [i.e., 40–55 Energy% (En%)], and include both n–3 and n–6 essential FAs: at least 50 and 300 mg/100 kcal ALA and LA, respectively (i.e., 0.45 and 2.7 En%). A guidance upper level of 1400 mg/100 kcal (12.6 En%) is provided for LA. While no maximum level was set for ALA, the LA/ALA ratio in formula is set to be between 5 and 15. For preformed DHA, Codex defines no minimum level and a guidance upper level of 0.5% of total FAs (22 mg/100 kcal, 0.2 En%) and specifies that the DHA content should be matched with at least equal levels of ARA. The guidance upper levels stated are based on an established history of apparent safe use, although several populations with a high fish intake (e.g., Japanese mothers) have far more DHA than ARA in HM (5). It is acknowledged, however, that there is not enough information for a science-based risk assessment to define strict maximum allowable levels (54, 55).

**FIGURE 1:**
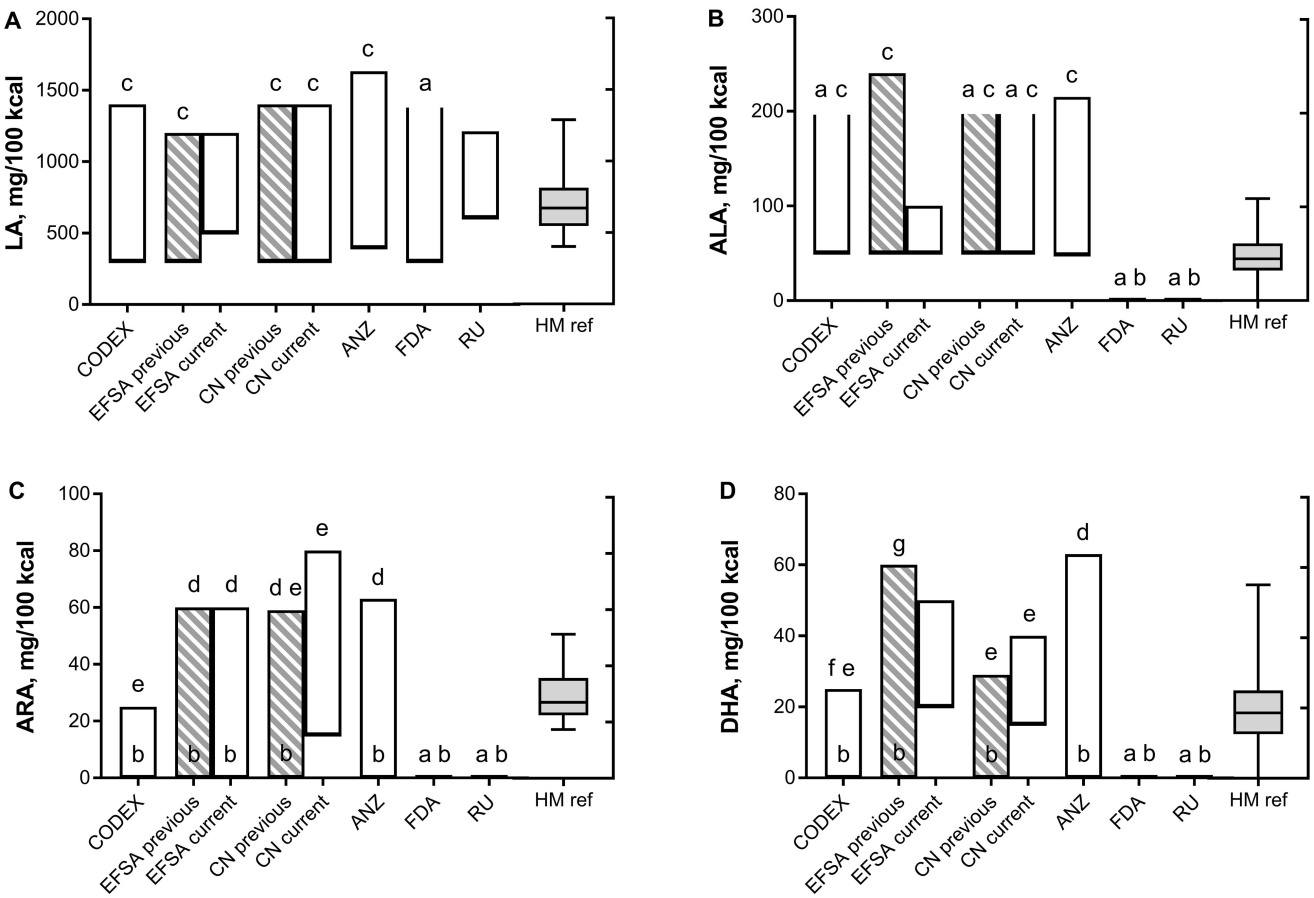
Boxes represent the minimum and maximum recommended/stipulated levels of LA, ALA, ARA, and DHA as set by Codex and various local regulatory bodies; values are expressed as mg/100 kcal and are calculated assuming 3.5 g fat/100 mL for infant formula. (A) No maximum level defined; (B) no minimum level defined (addition is not mandatory); (C) LA/ALA ratio needs to be between 5 and 15; (D) addition at maximum of 1% of total FA; (E) ARA/DHA ratio ≥1; (F) guidance upper level is 0.5% of total FA; and (G) shall not exceed n–6 LCPUFA. The box plot at right represents the median, upper, and lower quartiles and the range (minimum to maximum) in which these FAs are present in HM; values are expressed as mg/100 kcal and are calculated assuming 3.3 g fat/100 mL and using mean values in milk of mothers of term infants (average colostrum, transitional, and mature milk) that were reported in 50 studies (60 groups varying in size from 5 to 602 subjects) published between 1985 and 2018 that were included in a recent review on HM FA composition (2). Abbreviations: ALA, α-linolenic acid; ANZ, Australia New Zealand; ARA, arachidonic acid; CN current, China standards as published in February 2021 and mandatory from February 2023 onwards; CN previous, China standards before February 2021; EFSA current, European Food Safety Authority standards mandatory from February 2020 onwards; EFSA previous, European Food Safety Authority standards before February 2020; HM, human milk; HM ref, human milk reference; LA, linoleic acid; LCPUFA, long-chain PUFA; RU, Russia.

**FIGURE 2:**
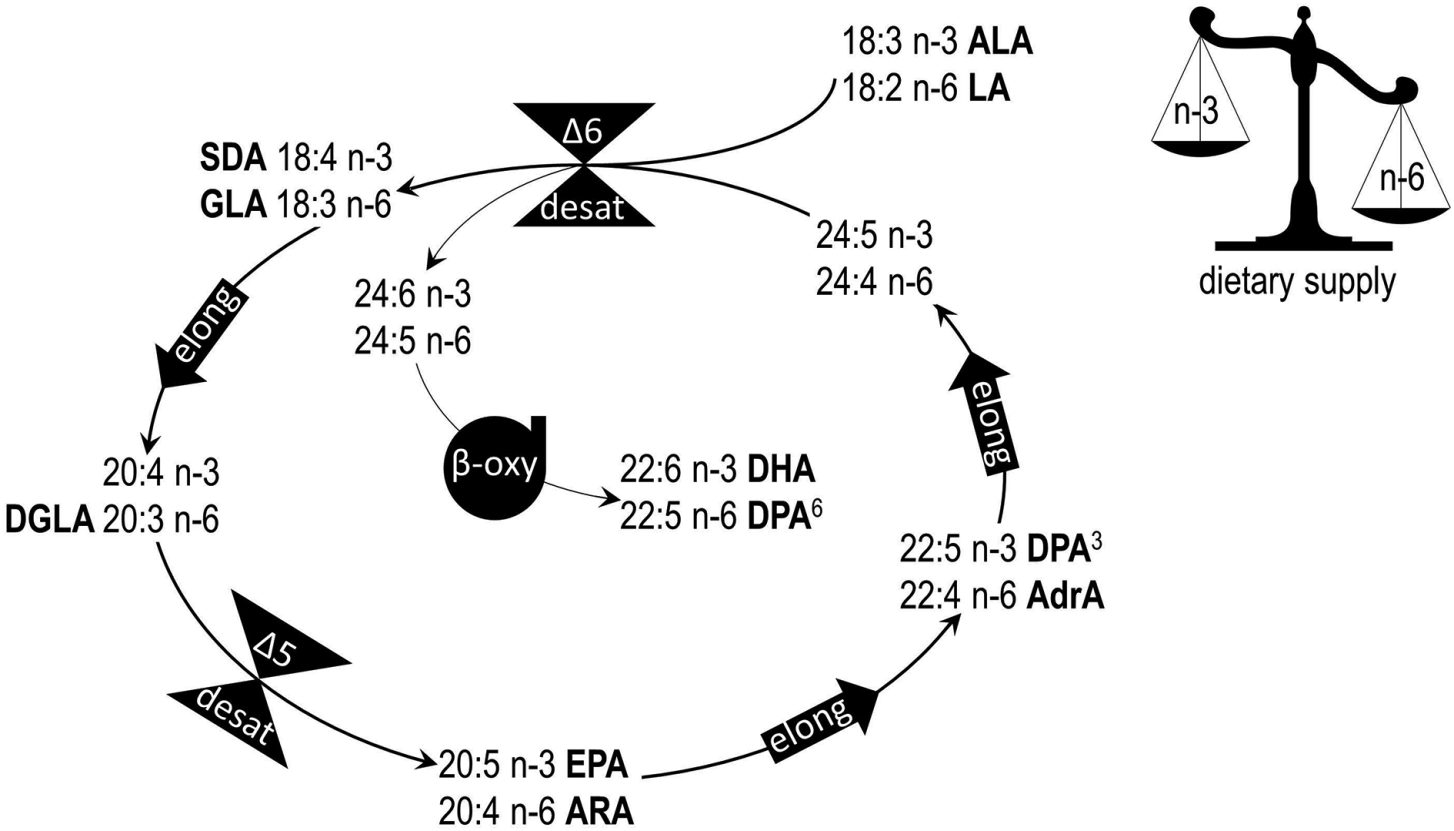
Metabolic pathways and interconversions of PUFAs. Adapted from Gibson et al. (57) with permission from Elsevier. Not shown are the metabolic steps required for activation of FAs to enter the pathways, and the steps required to accept synthetic products from the pathway. Abbreviations: AdrA, adrenic acid; ALA, α-linolenic acid; ARA, arachidonic acid; β-oxy, β-oxydation; DGLA, di-homo-γ-linolenic acid; desat, desaturase; DPA^3^, docosapentaenoic acid n–3; DPA^6^, docosapentaenoic acid n–6; elong, elongase; GLA, γ-linolenic acid; LA, linoleic acid; SDA, stearidonic acid.

The Codex recommendations (56) are partly based on data on the FA compositions of HM from healthy, omnivorous women, as well as observations from infant feeding studies (28, 55). The Codex represents guidelines on adequate infant nutrition that are meant to provide guidance for national and regional food standards. The Codex guidelines are implemented in derived legislation in most countries to ensure that infant formula is safe and meets the nutrient and energy requirements of developing infants. In addition, several national or regional authorities [e.g., the FDA in the United States, the European Food Safety Authority (EFSA) in the European Union, and Food Standards Australia New Zealand (ANZ) in Australia and New Zealand] provide guidance on infant formula compositions based on their own expert consultations. An overview of the recommendations by Codex and regulations set by various local authorities are provided for LA, ALA, ARA, and DHA in **Figure 1** , as well as the ranges of these FAs in HM. **Figure 2** in the Text Box illustrates the metabolic pathway for conversion of ALA and LA to their respective longer chain metabolites (57).

Global and national/regional standards are periodically updated with the intention of improving guidelines. Changes are based on emerging scientific insights on functional and compositional properties of FAs in nutrition and/or infant FA requirements. As an example of evidence-based adaptations to guidelines or regulations, during the last decades it became evident that an adequate nutritional supply of preformed DHA to infants has direct health benefits (58). As a result, the most recent European Commission (EC) directive (2016/127) (59) now prescribes the *mandatory* addition of preformed DHA to infant formula, as opposed to its *optional* addition in the previous versions of these guidelines. Conversely, and not in line with Codex recommendations, specific regulations on the addition of preformed ARA to formula (in conjunction with DHA) were deemed unnecessary, as it was considered that there was no conclusive evidence of functional effects of preformed ARA (59–61). This aspect of the European regulation is controversial. Of particular relevance to this paper is that having no ARA in formula results in an FA composition of formula deviating further from HM FA compositions, where both ARA and DHA are always present (5). In response to the release of the DHA recommendations, arguments were made by experts that the ARA content of infant formulas should be at least equal to that of DHA (55, 62). Following this reasoning, the addition of preformed ARA may allow for a decrease in the LA levels of formula.

## Evidence Base for the (Recently Adapted) LA and ALA Recommendations

Although >200 different FA species have been identified in HM, there is a tendency for some FAs to become the focus of intense scrutiny, including by committees and panels. As a result, other long-established constituents may not have been studied despite their potential functional relevance; in some cases, those constituents have never or have not recently been investigated or subjected to randomized controlled trials (RCTs). Constituents that have not received much attention in recent years include LA and ALA.

The minimal levels of LA set in the current guidelines are largely based on conclusions drawn from clinical experiments in the 1940s and 1950s that evaluated the essentiality of dietary LA intake in infants. Formulas very low in or devoid of any fat resulted in skin pathology (dryness and scaling) and in growth faltering in infants (28, 63–65). In preclinical models, LA deficiency resulted in severe impairments in renal function and fecundity in animals (64, 66, 67), all of which could be alleviated by providing LA. Minimal required levels of LA were found to be around 2 weight% (w%) of the total fat intake (i.e., 120 mg/100 kcal). It must be stressed, however, that these studies were done in the absence of ALA and a preformed LCPUFA supply. Interestingly, a recent report demonstrated that LA requirements in the presence of ALA are lower (1%–1.5%) than the historical 2% of energy intake value in rats, suggesting there may be a need to also reconsider the minimum LA recommendations for infants (68).

The maximal recommended LA intake, however, was less clear and rather arbitrarily set at a level seldom encountered in HM at that time, and based on experimental indications of possible untoward effects of high LA intakes and high dietary LA/ALA ratios. In the 1980s, a UK working group concluded that no obvious adverse effects were observed in infants raised on a formula with close to 60 w% LA of total fat (29), but the working group raised theoretical concerns with regard to the vitamin E requirements of the infants (31). Subcutaneous fat deposited in those infants was found to comprise 25 w% LA by 6 wk, which increased up to 46 w% at 12 wk. Red blood cell membrane phospholipids were found to contain increased LA levels in infants fed this formula compared to their breastfed peers. Other changes in the FA composition due to high LA dietary levels were hypothesized, but no clear short- or long-term harm was identified as long as the vitamin E supply was sufficient. The UK working group set a safe maximal LA level of 20% of the FA in a formula with 6 g fat/100 kcal, which equates to 1200 mg LA/100 kcal. This is the maximal level of LA set in the current European Union guidelines.

The essentiality of ALA has been established in animal studies only, leading to a recommended intake of 0.5% of the total caloric intake for infants (67). A clear role for ALA in infant development was never investigated, and clinical data on minimal requirements of ALA in infants, or on safe maximal intake levels, are lacking. Preclinical rodent data, similar to those which established LA and ALA as required in the absence of preformed LCPUFAs, show conclusively that diets with ARA and DHA and without any other source of n–6 or n–3 FAs are able to support normal growth and development, including cognitive development through 10 generations, and suggest improved fertility with advancing age compared to conventional soybean oil–based diets, which are high in LA and lacking ARA and DHA (4, 69). Based on these insights, LA and ALA may in fact not be essential as long as a sufficient level of preformed LCPUFA (DHA and ARA) is provided.

In the latest EC directive, the level of LA is set between 500 and 1200 mg/100 kcal (10%–25% of total FA) and the level of ALA is set between 50 and 100 mg/100 kcal (1%–2% of total FA) (59). Compared to the previous EC directive of 2006 (70), this stipulates a higher minimum addition of LA and a lower maximum of ALA (see Figure 1). The decision to lower the maximal allowed ALA levels might have been considered because of the now-mandatory addition of DHA. It was reasoned that adequate amounts of preformed DHA should be provided in the diet to meet infant requirements, allowing a lower level for its precursor ALA. The basis of this seems to be that whereas ALA can function as a precursor for DHA (see **Text box**), the endogenous conversion of ALA to DHA is regarded as insufficient during infancy to meet DHA requirements, and is also known to depend on the infant's genotype with respect to the desaturase and/or elongase enzymes required for ALA to DHA conversion (9, 42, 43). The higher levels of LA in the new guidelines (a higher absolute minimum, as well as a higher LA/ALA ratio due to the lowered maximum addition of ALA) are thought to facilitate LA conversion to ARA, given that the addition of preformed ARA is not proposed (71). While this logic may seem sound (see Text box), whether adequate ARA levels can be achieved from LA conversion to meet the high metabolic demands of growing infants has been questioned (72). Interestingly, although a higher LA supply might theoretically favor endogenous ARA synthesis (see Text box), there is evidence coming from studies in adults that this relationship is absent or much more complex (73). As an example, targeted dietary manipulations in adults showed that a 66% reduced LA intake (from 7.4 to 2.4 En%), along with a 50% reduced preformed ARA intake, only resulted in a 5% reduction (borderline significant) in circulating ARA levels (74). This indicates that circulating plasma ARA levels neither show a linear association with nor are a mere reflection of the dietary LA or ARA intake, but appear to depend on endogenous production (capacity) aimed at maintaining physiologically adequate levels. Moreover, it should be noted that high dietary LA levels may suppress endogenous synthesis and tissue accretion of all n–3 FAs (see Text box), thus in effect creating a metabolic requirement for preformed DHA (75, 76). Conversely, a lower dietary LA supply would theoretically allow for lower levels of the DHA supply (74, 77).

Text Box Human milk and infant formula include the essential fatty acids (EFAs) LA (18:2 n–6) and ALA (18:3 n–3). After ingestion, these 18-C EFAs can be converted to LCPUFAs (≥20-C), including n–6 ARA and n–3 DHA, by desaturase and elongase enzymes (see Figure 2); this process takes place primarily in the liver, although in infants the capacity to synthesize LCPUFAs from EFAs is low (8, 71). HM also contains preformed LCPUFAs (both n–6 and n–3), and so may infant formula.

The n–6 and n–3 PUFAs use the same set of enzymes, and therefore compete for conversion. Consequently, the absolute amounts of both PUFA type, as well as their ratio, affect the balance between n–6 and n–3 LCPUFA synthesis. A high supply of LA can limit n–3 LCPUFA synthesis and lead to excessive n–6 LCPUFA synthesis. The conversion of EFAs, as well as of the intermediates n–6 AdrA and n–3 DPA^3^ to n–6 DPA and DHA, respectively, involves the *FADS2*-coded enzyme Δ6-desaturase via either the Sprecher pathway or *FADS2*-coded Δ4-desaturase (78, 79). A high supply of LA can therefore also limit the conversion rates of n–6 and n–3 FA intermediates.

LA and ALA, as well as their metabolites, are important constituents of biological membranes and immune-modulating compounds, as they are precursors in the biosynthesis of eicosanoids, signaling lipidic molecules with an important function in the immune system (allergy and inflammation) and in adipose tissue development (80). The n–6 and n–3 PUFAs compete for uptake from the plasma to organs, such as the developing brain, where ARA and DHA cannot be synthesized locally in sufficient amounts (81). A disproportionally high supply of LA over ALA will be reflected in a disbalance in the types of end products, including LCPUFAs, eicosanoids, and PGs, synthesized from the precursors supplied and in their availability for incorporation in and/or use by organs and tissues. Although circulating PUFA levels do not necessarily reflect local tissue levels, a high circulating n–6 PUFA status may inhibit the local uptake and incorporation of (preformed) n–3 LCPUFAs in membranes (77, 82, 83). The dietary supply with n–3 LCPUFAs for health benefits may therefore not be effective when combined with an imbalanced intake of LA/ALA (71).

## Potential Effects of a High Dietary LA Intake in Early Life

Many organs and systems undergo important developmental steps, particularly during the first 1000 d from conception to a child's second birthday. The dietary FA composition in the early postnatal period was proposed to modulate growth and development, and ultimately to affect health in later life (84). Moreover, it was postulated that an increased dietary LA intake and a decrease in the ALA supply early in life might have negative effects on short- and long-term health. The changes in human dietary habits over recent decades in Western society, including higher intakes of LA, have coincided with higher incidences of obesity, immune-related diseases, and neuropsychiatric diseases at a population level (3, 84–89). Although it is important to note that an association does not imply any causal relationship, this has nevertheless led to speculation that exposure to higher LA intakes before birth and in early infancy might be associated with altered development and long-term consequences for health and disease risks. The increased dietary LA supply over the last decades in Western populations is reflected in an increasing mean HM LA content (3). Some observational studies propose that high LA in HM might be associated with poor neurocognitive outcomes (90–93), excessive weight gain and a higher obesity risk (50, 94), and an increased risk for atopic eczema and allergic responses (95–97), although there is no direct clinical evidence supporting a causal relationship. LA and ALA are precursors for n–6 and n–3 LCPUFAs, respectively, as well as a wide range of other metabolites and bioactive compounds (see Text box). Dietary intakes of LA and ALA, and the balance between them, therefore have the potential to affect the LCPUFA status, and thus impact immune and neural functions, as well as the development of adipose tissue, particularly if preformed LCPUFAs are not adequately provided. The following paragraphs elaborate on the evidence base for this hypothesis.

### LCPUFA status

Circulating n–3 LCPUFA plasma levels in infants are strongly affected by the dietary supply of preformed n–3 LCPUFAs (6, 45). However, intervention studies in humans where the dietary n–6/n–3 ratio is modified by adapting only the levels of the precursors LA and ALA are scarce. The evidence from available trials in adults suggests that the n–3 LCPUFA status may be increased by specifically reducing dietary LA intakes to very low levels (74, 76, 98–101). Udell and colleagues (102) reviewed the outcomes of clinical intervention studies in infants using formula with an increased ALA level, thereby also resulting in lower dietary LA to ALA ratios, and concluded that an increasing ALA supply improved the infant DHA status, in contrast to many studies in adults (8). In line with these findings, the infant DHA status was also increased when the dietary ALA supply was increased while LA was lowered (103, 104). Importantly, these studies in infants were performed using formulas devoid of preformed LCPUFAs, and the ALA levels were substantially above the current recommended range for ALA. With the mandatory presence of preformed DHA in current infant formulas, the contribution of ALA as precursor to DHA is likely to be limited, since preformed DHA consistently raises plasma DHA levels (8, 71, 78, 105).

### Immune function and allergy

The immune system develops early in life. LA and ARA have been proposed to induce pro-inflammatory effects because of the range of pro-inflammatory eicosanoid mediators (e.g., PGs, leukotrienes, see Text box) derived from LA and ARA by cyclo- and lip-oxygenases (80, 106, 107). The levels of these PUFAs, and therefore their downstream products, are postulated to play a role in processes such as inflammation and thrombosis and, as suggested in more recent studies, influence the response to viruses, including severe acute respiratory syndrome coronavirus 2, that are known to be related to such signaling molecules (78). However, lipoxins derived from ARA may also induce resolution of inflammation. A high dietary supply of LA was proposed to facilitate development of allergic sensitization, and it was speculated that enhanced production of the ARA-derived eicosanoid PGE_2_ might induce multiple T helper 2 cell–associated diseases, most notably atopic dermatitis and asthma (108–110). PGE_2_ might also promote the production of IgE, an immunoglobulin associated with allergic responses (110). Furthermore, LA might affect signal transduction pathways involved in immune functions by binding to transmembrane and/or intracellular receptors or altering other signaling molecules (48). While higher intakes of LA-rich vegetable oils, such as conventional soybean oil, have been associated with an increased incidence of allergic disease in children (111), confounding of the association with other variables associated with allergy risks must be considered. In preclinical models, excessive dietary LA intake promotes vascular inflammation (112) and is associated with allergic responses (113, 114), but it is not clear whether these findings can be extrapolated to human infants.

### Neurocognitive function

Early in life, structural and functional development of the brain is enabled by high levels of DHA and other substrates sourced from the blood stream and accumulating in neuronal tissues. In the absence of an adequate preformed DHA supply, a high dietary LA supply might reduce the brain DHA uptake, since *1*) the capacity for endogenous n–3 LCPUFA synthesis will most likely be reduced by high LA levels competing with ALA for conversion; and *2*) higher n–6 LCPUFAs in the circulation compete with n–3 LCPUFAs for incorporation into neuronal membranes, resulting in relatively lower levels of DHA and other n–3 LCPUFAs in the brain [see for review (115)]. Although brain FA profiles cannot be studied in clinical trials, autopsy studies in infants that suffered from sudden (cot) death revealed differences depending on diet. Lower brain DHA and higher n–6 docosapentaenoic acid (DPA) contents were observed in infants fed formulas with a relatively high LA content and no preformed DHA compared to both breastfed infants and formula-fed infants exposed to a high LA/ALA ratio (116–119). Evidence from preclinical studies shows that postnatal dietary LA levels are inversely related to the DHA content in the developing brain. In contrast, the level of, in particular, n–6 DPA, which has no neurodevelopmental relevance and typically accumulates if there is an n–3 PUFA deficiency (120, 121), is also increased following a high LA supply (82, 83, 122, 123). Supplementation with DHA and ARA did not prevent some of these typical changes in the brain FA profile caused by the high LA supply (122). In contrast, specifically lowering the LA supply early in life was shown to positively affect structural development of the brain and to improve neurocognitive function later in life in experimental animal models (124, 125).

### Adipose tissue development and metabolic syndrome

White adipose tissue (WAT) development occurs early in life and comprises proliferation and differentiation of preadipocytes to mature adipose cells. In humans, the WAT storage capacity—the number of adipocytes that can be filled throughout life—is established during childhood and adolescence (126). The n–3 and n–6 FAs may differentially modulate proliferation and differentiation of preadipocytes, with LA and ARA and their eicosanoid metabolites stimulating adipogenesis via several mechanisms, including gene transcription, mRNA processing, and posttranscriptional processes (3, 127, 128). Data from preclinical studies suggest that exposure to a high LA supply early in life predisposes an infant to later life metabolic diseases, including obesity and hepatic steatosis (3, 127, 129–131), whereas low LA intake, as well as dietary n–3 LCPUFA supplementation, may program an infant toward reduced fat mass accumulation (132, 133). It is important to note, however, that this has not been a consistent finding across all preclinical studies, and the interpretations of these data are often complicated by the fact that exposure to the low-LA or high-DHA diet is not confined only to the preweaning period (134). While positive associations between cord blood n–6 PUFAs and fat mass in childhood have been reported in human studies, no causal relationships have been established (135). Although there is some indication that HM DHA levels may be associated with beneficial effects on childhood BMI (136), the potential for reducing fat deposition (in adipose tissue) in human infants through n–3 FA supplementation during pregnancy and/or breastfeeding is not supported by the current body of evidence (137–139).

## Gaps in Knowledge

The latest EC directive on infant and follow-on formula compositions stipulated higher minimal LA and lower maximal ALA contents, with a possible increase of the LA/ALA ratio, in the presence of DHA and potentially also ARA. Based on experimental and observational data described in the previous sections, potential adverse effects of these changes are conceivable and should be carefully explored and considered. However, the complexity of clinical intervention trials in general has posed a challenge for generating sufficient evidence for the specific contributions and optimal levels of distinct FAs in infant nutrition, such as LA, ARA, and ALA. For example, measurable clinical endpoints may stretch well beyond the end of any intervention study. Although several clinical trials have investigated the effects of a high compared with low n–6/n–3 ratio early in life on infant development and health outcomes, most studies focus on supplies of preformed n–3 and n–6 LCPUFAs. To the best of our knowledge, there are no clinical trials to date in which the effects of LA and ALA levels in infant formula are studied in the presence of preformed LCPUFAs. This is critical given that the available preclinical evidence indicates that the relationship between n–6 and n–3 FA intakes and circulating DHA levels is more complex than the simple concept of a ratio and also depends on the total dietary PUFA provided.

## Rationale and Hypothesis Behind Health Benefits of Lowering LA Levels in Formula

Based on our current understanding of lipid biochemistry and functionality and on mainly preclinical evidence, it can be postulated that a disproportionally high LA intake in infants may reduce n–3 LCPUFA synthesis and/or accretion, resulting in a lower DHA status (in the absence of preformed DHA). Conversely, the synthesis of n–6 LCPUFA–derived pro-inflammatory eicosanoids and adipogenic cytokines might be increased, with a potential impact on the development and functioning of the immune system, brain, adipose tissue, and other organs. It can be hypothesized that lowering the LA content in infant formula, without changing ALA, DHA, and ARA levels, might support an enhanced infant n–3 LCPUFA status and thereby support healthier infant development. Potentially, this might lead to further reconsideration of the level of preformed DHA needing to be added to formula. This hypothesis may have considerable relevance for long-term health outcomes, and hence should be thoroughly explored.

## Developing a Better Evidence Base for Adequate and/or Optimal LA Intakes in Infants

To create clarity on optimal and safe LA levels in infant formula intended for healthy, term infants, as well as to generate convincing evidence for potential health benefits, including the possible reduction of disease risks later in life, more clinical evidence is needed. For example, as a first step, a focused proof-of-principle study could be performed to show the impact of an infant formula with low compared with high (current) LA levels on the LCPUFA status in healthy, term infants. Such a study could be done as a relatively small RCT, using potentially an infant formula with a maximal LA content of 300 mg/100 kcal (i.e., the minimum level defined in the previous EFSA recommendations and current Codex) and a formula containing at least 500 mg/100 kcal LA (in line with current EFSA recommendations). In order to provide valid data, both the experimental (low LA) and control (high LA) formulas should contain similar ALA levels, as well as preformed DHA and ARA, the latter preferably in equal amounts (i.e., around 25 mg/100 kcal), following recently described recommendations (55). In addition, the experimental or control formula should be the sole source of nutrition up to at least 4 months of age. The study may include a breastfed reference group, as well as a group fed a formula low in LA but without ARA, to evaluate the impact of the absence of preformed n–6 LCPUFAs, as allowed by current EFSA recommendations. Crucial in such a study will be the definition of the LCPUFA status and the determination of how to assess this optimally. Plasma and erythrocyte FA levels, measuring both precursor FAs and LCPUFAs (140), could be used to monitor the FA status over time. Such a proof-of-principle study should be conducted in a well-defined and fairly homogenous population to reduce the impact of potential sources of variation, as mentioned above (e.g., genotype, phenotype, and maternal environmental factors). It is important to note that this has the inherent yet acceptable limitation that the outcomes cannot be directly extrapolated to other (sub-)populations, including preterm infants.

The proposed adaptations of the infant formula recipes to be tested in the trial may raise additional challenges that will impact the overall FA composition of infant formula, since lowering the LA content requires an increase in another FA to keep the total contribution of fat equal. Using HM as guidance in how to do this, a higher contribution of oleic acid or a saturated FA might be the most logical solution. Although also increasing the ALA content may be considered due to ALA's role as precursor for n–3 LCPUFA, preclinical evidence in rats shows that endogenous DHA synthesis may be paradoxically reduced when the ALA supply is too high (141).

Based on the established clinical evidence that associates the infant blood FA status with functional outcomes, emphasizing brain developmental milestones and immune function, it may be assumed that within reasonable ranges a higher n–3 LCPUFA status could be beneficial to the developing infant (142). This should be confirmed in follow-up clinical studies that will also consider functional outcomes, such as growth; body composition; the incidence of atopic disease; neurodevelopment, including vision development; and cognition in the long term. Whereas the effects of dietary LCPUFA intake after weaning will contribute to the outcomes, concrete clinical endpoints would preferably be assessed until an age of 2 y (∼1000 d) and with follow-up beyond (at 5 or even 10 years of age). Such data could provide the required new evidence that is needed to reconsider current guidelines for the level of LA in formula for healthy, term infants and may help define levels that will support healthier development of infants across the population.

## Conclusion

The balance of dietary LA and ALA levels and preformed n–6 and n–3 LCPUFAs in early life nutrition has the potential to affect the LCPUFA status and impact immune, neural, and adipose tissue development. The latest EC directive on infant formula compositions stipulated increasing the minimal LA content and lowering the maximal ALA content in infant formula, thereby increasing the LA/ALA ratio, in the presence of DHA and optionally also ARA. Based on the current understanding of lipid biochemistry and on the available scientific, mainly preclinical, evidence, it may be postulated that a relatively high LA intake in infants could reduce n–3 LCPUFA synthesis and/or accretion, resulting in a lower DHA status. However, the available preclinical evidence indicates that the relationship between n–6 and n–3 FA intakes on circulating DHA levels is more complex, and is not only the direct result of the LA/ALA ratio but also depends on the total (preformed) dietary PUFAs provided. We conclude that a clear gap in knowledge exists regarding the potential impacts of LA and ALA levels in infant formula in the presence of preformed LCPUFAs, as in current formulas. Hence, an urgent need exists for well-designed clinical intervention trials to create clarity about optimal and safe levels of LA and its long-term implications on functional health outcomes.

## Notes

The current paper is the outcome of a series of discussion sessions initiated and facilitated by Danone Nutricia Research (DNR), in which all coauthors actively participated. SEC, JTB, CA, PCC, SF, PL, BVK, and BM received an expense allowance from (DNR) to compensate for their effort.

Author disclosures: SEC, JTB, CA, PCC, SF, PL, BVK, and BM received an expense allowance from Danone Nutricia Research (DNR) to compensate for their effort. LS, MA-B, and BJMvdH are employed by DNR. EMvdB was employed by DNR at the time of the topic discussions. BVK is the Else Kröner Senior Professor of Paediatrics, a position cofunded by the Else Kröner–Fresenius Foundation, Bad Homburg, Germany, and Ludwig Maximilian University of Munich Hospitals.

Danone Nutricia Research contributed to the discussion, the interpretation of the outcomes, and the design of the paper.

Perspective articles allow authors to take a position on a topic of current major importance or controversy in the field of nutrition. As such, these articles could include statements based on author opinions or point of view. Opinions expressed in Perspective articles are those of the author and are not attributable to the funder(s) or the sponsor(s) or the publisher, Editor, or Editorial Board of *Advances in Nutrition*. Individuals with different positions on the topic of a Perspective are invited to submit their comments in the form of a Perspectives article or in a Letter to the Editor.

Abbreviations used: ALA, α-linolenic acid; ARA, arachidonic acid; DPA, docosapentaenoic acid; EC, European Commission; EFA, essential fatty acid; EFSA, European Food Safety Authority; FA, fatty acid; *FADS*, fatty acid desaturase; HM, human milk; LA, linoleic acid; LCPUFA, long-chain PUFA; RCT, randomized controlled trial; WAT, white adipose tissue.
